# Combined hyperforin and lanicemine treatment instead of ketamine or imipramine restores behavioral deficits induced by chronic restraint stress and dietary zinc restriction in mice

**DOI:** 10.3389/fphar.2022.933364

**Published:** 2022-08-24

**Authors:** Bartłomiej Pochwat, Paulina Misztak, Julia Masternak, Ewa Bączyńska, Krystian Bijata, Matylda Roszkowska, Monika Bijata, Jakub Włodarczyk, Małgorzata Szafarz, Elżbieta Wyska, Bożena Muszyńska, Agata Krakowska, Włodzimierz Opoka, Gabriel Nowak, Bernadeta Szewczyk

**Affiliations:** ^1^ Department of Neurobiology, Maj Institute of Pharmacology Polish Academy of Sciences, Krakow, Poland; ^2^ Laboratory of Cell Biophysics, Nencki Institute of Experimental Biology, Warszawa, Poland; ^3^ Faculty of Chemistry, University of Warsaw, Warszawa, Poland; ^4^ Department of Pharmacokinetics and Physical Pharmacy, Jagiellonian University Medical College, Kraków, Poland; ^5^ Department of Pharmaceutical Botany, Pharmacy Faculty, Jagiellonian University Medical College, Kraków, Poland; ^6^ Department of Inorganic and Analitycal Chemistry, Pharmacy Faculty, Jagiellonian University Medical College, Kraków, Poland

**Keywords:** refractory depression, ketamine, hyperforin and lanicemine, zinc deficiency, chronic stress

## Abstract

Clinical and preclinical studies show evidence that chronic stress or nutritional deficits in dietary zinc (Zn) intake may be risk factors for developing major depressive disorder (MDD). Furthermore, there may be possible links between low serum Zn levels and development of treatment-resistant depression. In the present work, we combined chronic restraint stress (CRS) and a low-zinc diet (ZnD) in mice and carried out a set of behavioral and biochemical studies. The mice were treated with four different antidepressant compounds, namely, ketamine, Ro 25–6981 (Ro), hyperforin and lanicemine (Hyp + Lan), and imipramine (IMI). We show that CRS or ZnD alone or a combination of CRS and ZnD (CRS + ZnD) induces anhedonia observed in the sucrose preference test (SPT). The behavioral effects of CRS were restored by ketamine or IMI. However, only Hyp + Lan restored the deficits in behavioral phenotype in mice subjected to CRS + ZnD. We also showed that the antidepressant-like effects observed in Hyp + Lan-treated CRS + ZnD mice were associated with changes in the morphology of the dendritic spines (restored physiological level) in the hippocampus (Hp). Finally, we studied the metabolism of ketamine and its brain absorption in CRS and CRS + ZnD mice. Our results suggest that CRS + ZnD does not alter the metabolism of ketamine to (2R,6R;2S,6S)-HNK; however, CRS + ZnD can induce altered bioavailability and distribution of ketamine in the Hp and frontal cortex (FC) in CRS + ZnD animals compared to the control and CRS groups.

## Introduction

MDD is a severe public health problem that affects over 264 million people worldwide and is also a significant cause of disability (GBD 2017 Disease and Injury Incidence and Prevalence Collaborators, 2018; Cruz-Pereira et al., 2020). Although MDD is clinically defined as a single disease, it is diagnosed by the occurrence of different symptoms with different biological bases, indicating the heterogeneous nature of the disorder. The differentiation of the clinical symptoms and their biological substrata is reflected in the complex pathogenesis and etiology of MDD. In other words, there is no single crucial cause that induces a complex disorder such as MDD (Malhi and Mann, 2018). Improper lifestyle, environmental, and genetic factors are often involved in the development of MDD symptoms; a considerable amount of evidence (clinical and preclinical) indicates that chronic stress and nutritional errors may also be critical variables in the etiology of MDD (Yang L. et al., 2015; Szewczyk et al., 2018; Antoniuk et al., 2019; Park et al., 2019). For example, decreased dietary Zn intake could be a risk factor for developing MDD. Zn deficiency in humans is an emerging global health concern that is estimated to affect approximately two billion people globally (Hussain et al., 2022). Inadequate Zn supply coupled with its poor bioavailability in the diet and excessive loss during diarrhea account for the primary causes of Zn deficiency (Maares and Haase, 2020; Hussain et al., 2022). Some epidemiological studies have suggested a relationship between low dietary Zn intake and MDD (Jacka et al., 2012; Vashum et al., 2014). Other reports have revealed a negative correlation between the serum Zn levels and severity of MDD symptoms (Hansen et al., 1983; Maes et al., 1997); decreased serum Zn levels have also been reported in people suffering from MDD (Marcellini et al., 2006; Lehto et al., 2013; Siwek et al., 2013; Markiewicz-Zukowska et al., 2015). The role of Zn level deficit in inducing MDD symptoms has also been demonstrated in preclinical paradigms; in these studies, rats and mice chronically fed with a ZnD exhibited depression-like symptoms in a set of behavioral tests (Whittle et al., 2009; Mlyniec et al., 2012; Mlyniec and Nowak, 2012; Doboszewska et al., 2015a; Doboszewska et al., 2015b). Similarly, rodents fed a low-zinc diet exhibited a depression-like behavioral phenotype similar to rats and mice subjected to chronic stresses. Chronic stressed and ZnD rodents were characterized by anhedonia (measured by SPT or sucrose intake test (SIT)), increased immobility time (forced swimming test or tail suspension test (TST)), or altered social behaviors (Whittle et al., 2009; Li et al., 2011; Castro et al., 2012; Mlyniec et al., 2012; Mlyniec and Nowak, 2012; Pochwat et al., 2014; Doboszewska et al., 2015a; Doboszewska et al., 2015b; Yang et al., 2018). Moreover, the efficacy of classic monoamine-based (chronic administration) or atypical (fast-acting compounds, such as ketamine) antidepressants are similar in the two preclinical paradigms (Li et al., 2011; Doboszewska et al., 2015b; Pochwat et al., 2020). The biochemical effects induced by chronic stress and ZnD in rodents do not fully overlap (Li et al., 2011; Pochwat et al., 2020). We emphasize that not much is known about the behavioral and biological effects induced by a combination of chronic stress and ZnD in rodents. The key to understanding the etiology, pathogenesis, and pharmacology of MDD lies in unraveling the behavioral and biological effects of CRS and ZnD; this is because some clinical data have clearly shown that reduced serum Zn levels and lack of normalization thereof after classic antidepressant administration in patients with refractory depression support the potential association between disturbances in Zn levels and efficacies of classic antidepressants (Maes et al., 1997; Maes et al., 1999; Siwek et al., 2010). The possible relationship between reduced serum Zn levels and treatment-resistant depression is only indicated for monoamine-based antidepressants. Not much is known about the serum Zn levels and potency of novel antidepressant compounds, such as ketamine. Therefore, we wished to investigate whether a combination of risk factors such as chronic stress and dietary zinc restriction could induce resistance to atypical antidepressants such as ketamine, Ro, and a combination of Hyp + Lan (Pochwat et al., 2018), as well as the classic antidepressant IMI. We believe that the effectiveness of ketamine in these experimental conditions would be very interesting as it is considered a pharmacological weapon against treatment-resistant depression with an estimated efficacy of 50–70% (Serafini et al., 2014). Additionally, the biological factors that induce lack of responsiveness to ketamine in monoamine drug-resistant depression are unknown. In our first series of experiments, we assessed the behavioral alterations in mice subjected to a combination of CRS + ZnD. Based on the results of the behavioral studies, we conducted molecular and anatomical experiments (analysis of dendritic spine remodeling and density) to identify the specific biological changes underlying resistance to antidepressants in CRS + ZnD mice.

## Materials and methods

### Animals

Experiments were conducted using 7-week-old male C57BL/6J mice (Charles River, Germany) housed under standard laboratory conditions of lighting (light phase: 7:00–19:00) and temperature (19–21 °C), with free access to water and food. The control and stressed animals were weighed twice a week. Similar to the control group, the stressed mice were housed six per cage. All experiments were conducted in accordance with the EU directive 2010/63/EU and with the approval of the local ethics commission (17/2019, 190/2019, 14/2020, 77/2020). All efforts were made to minimize animal suffering and reduce the number of animals used.

### Compounds and treatment

All the compounds/drugs used in the study were obtained commercially, except for hyperforin (sodium salt), which was received as a gift from Dr. Wilmar Schwabe GmbH and Co (Karlsruhe, Germany). The doses of the compounds were chosen based on results from our preliminary studies (ketamine, Ro, Lan, and Hyp) or literature (IMI). All compounds were dissolved in 0.9% NaCl and administered as follows: ketamine 10 mg/kg; Ro 10 mg/kg; Lan 10 mg/kg; IMI 10 mg/kg; and Hyp 2.5 mg/kg at volume doses of 10 mL/kg body weight. The control group received only 0.9% NaCl. The time and frequency of administration of these compounds are detailed in the schemes provided in the figures.

### Zinc deficiency

One week after acclimation, the mice were subjected to either a zinc-adequate feed (ZnA, 50 mg/kg) or zinc-deficient/low-Zn feed (ZnD, 3 mg/kg). Beginning from the second week, some ZnD mice were additionally subjected to 3 weeks of CRS. At the beginning of the 5th week, the mice were subjected to their appropriate treatment schedules or behavioral tests (see schemes in the figures).

### Chronic restraint stress

CRS was performed in accordance with the procedures of Ding et al. (2016) with modifications. Mice from the stress groups were restrained in well-ventilated tubes with caps and holes for breathing (in a separate room from the control groups) for 3 h/day over 3 weeks. After each restraint period, the mice were transferred to housing cages. The control mice were housed at 6 mice per cage in an enriched environment and were handled every third day.

### Sucrose preference test

Seven days before the tests, the control and stressed mice were singly housed. The tests were carried out as follows. Two bottles (water and 1% sucrose solution) were placed in each cage 63 h before the test. Twenty-four hours later, the bottles were switched from right to left. Fifteen hours before the tests, the animals were deprived of food, water, and sucrose. The tests lasted 2 h, and sucrose preference was calculated as a percentage of the volume of sucrose intake over the total volume of fluid intake (water and sucrose solution). The SPT was performed during the light phase.

### Tail suspension test

The TST was performed as described in a previous work (Pochwat et al., 2018). Briefly, each mouse was suspended individually by the tail using adhesive tape (2 cm from the tail tip) affixed to a solid flat surface, and immobility times were measured for 6 min. A mouse is considered to be immobile when it is suspended passively and is completely motionless.

### Pharmacokinetic analysis

The pharmacokinetic parameters of ketamine and its active metabolite hydroxynorketamine were estimated using noncompartmental analysis. The maximum serum and brain structure concentrations (C_max_) as well as the durations to arrive at these concentrations (t_max_) following intraperitoneal (i.p.) administration were obtained directly from the concentration versus time curves. The terminal slope (λ_z_) was obtained by linear regression, and the terminal half-life (t_0.5_λ_z_) was calculated as ln2/λ_z_. The area under the concentration–time curve from the time of dosing to infinity (AUC_0-∞_) was calculated using the linear trapezoidal rule. The extrapolated terminal area was assessed as C_n_/λ_z_, where C_n_ is the last measured data point. The volume of distribution based on the terminal phase (V_z_/F) and clearance divided by the fraction absorbed (CL/F) were calculated for the serum only according to equations D/(λ_z_·AUC_0-∞_) and D/AUC_0-∞_, respectively, where D is the dose. The mean residence time (MRT) was calculated as AUC_0-∞_/AUMC_0-∞_, where AUMC_0-∞_ is the area under the first moment curve from the time of dosing to infinity.

### Analytical method

Concentrations of ketamine and hydroxynorketamine in the plasma, FC, and Hp homogenates were measured by liquid chromatography tandem mass spectrometry (LC-MS/MS) using a Sciex QTRAP 4500 triple quadrupole mass spectrometer coupled to an ExcionLC AC high-performance liquid chromatography system (both from Danaher Corporation, USA). Stock solutions of ketamine and hydroxynorketamine (1 mg/mL) were prepared in methanol. Working standard solutions were also prepared in methanol by serial dilution of the mixed stock solutions at the following concentrations: 0.005, 0.01, 0.1, 0.25, 0.5, 1, 2.5, 5, 10, and 20 μg/mL. Samples for the calibration curve were prepared by spiking 45 μL of the matrix (plasma or tissue homogenate) with 5 μL of the standard working solution at the appropriate concentration level and vortexed for 10 s. The calibration curve concentrations were 1, 10, 25, 50, 100, 250, 500, 1000, and 2000 ng/mL of plasma or 0.005, 0.01, 0.1, 0.25, 0.5, 1, 2.5, 5, and 10 μg/g of brain tissue. The brain structures were homogenized in distilled water (1:9, *w*/*v*) using a handheld pestle and glass tube homogenizer (Potter-Elvehjem PTFE, Sigma Aldrich). The plasma or sample homogenates (50 μL) were deproteinized at a ratio of 1:3 with 0.1% formic acid in acetonitrile containing an internal standard (IS), briefly vortexed, and then centrifuged for 10 min at 8000 rpm (Eppendorf miniSpin centrifuge). The supernatant was transferred to autosampler vials, and samples with concentrations above the ULOQ were diluted 10 times with an appropriate matrix (plasma or tissue homogenate) and reanalyzed. Chromatographic separation was performed using an XBridge^®^ HILIC analytical column (2.1 × 150 mm, 3.5 µm, Waters, Ireland) at an oven temperature of 40 °C for 3 min. The autosampler temperature was maintained at 15 °C while a sample volume of 3 μL was injected into the LC-MS/MS system. The mobile phase containing 0.1% formic acid in acetonitrile in water was mixed at a ratio of 25:75 and supplied at 0.4 mL/min. Electrospray ionization (ESI) in the positive-ion mode was used for ion production. The mass spectrometric conditions and ion path parameters, such as collision energy (CE), declustering potential (DP), and entrance potential (EP), were optimized for each analyte by continuous infusion of the standard solution at the rate of 7 μL/min using a Harvard infusion pump. The tandem mass spectrometer was operated at unit resolution in the selected reaction monitoring (SRM) mode to observe the transition of the protonated molecular ions *m/z* 238 to 207 (CE = 20 V; DP = 30 V; EP = 10 V) and *m/z* 238 to 125 (CE = 38 V; DP = 30 V; EP = 10 V) for ketamine; *m/z* 240 to 115 (CE = 70 V; DP = 55 V; EP = 10 V) and *m/z* 240 to 151 (CE = 30 V; DP = 55 V; EP = 10 V) for hydroxynorketamine (the first pair was used as a quantifier and the second was used as a qualifier); and *m/z* 242 to 129 (CE = 35 V; DP = 76 V; EP = 10 V) for d_4_-ketamine (IS). The ion-source temperature was maintained at 500 °C, and the ion spray voltage was set to 4000 V. The curtain gas (CUR) was set at 35 psi and collision gas (CAD) at medium. The data acquisition and processing were accomplished using the Applied Biosystems Analyst version 1.7 software (Sciex, Framingham, MA, USA). The calibration curves were constructed by plotting the ratio of the peak area of the studied compound to IS versus drug concentration and generated by a weighted (1/x ⋅x) linear regression analysis. The validated quantitation ranges for this method were from 1 to 2000 ng/mL for plasma and from 0.005 to 10 μg/g for brain tissue, with accuracies of 94.27–106.95% and 95.13–102.56% for the plasma and brain, respectively. No significant matrix effects were observed, and there were no stability-related problems during the routine analysis of the samples.

### DiI labeling and morphometric analysis of dendritic spines

Brain tissue was fixed in neutral phosphate-buffered 4% paraformaldehyde solution for 1 h. The brains were immediately cut into 140-µm-thick slices with a vibratome. Then, to visualize the changes in the shape of the dendritic spines, the brain slices were subjected to random dendrite labeling using 1.6 µm tungsten particles (Bio-Rad, Hercules, CA, USA) coated with the propelled lipophilic fluorescent dye 1,1′-dioctadecyl-3,3,3,3′-tetramethylindocarbocyanine perchlorate (DiI; Invitrogen) delivered via gene gun bombardment (Bio-Rad). Images of the secondary dendrites of the neurons in the Hp and prefrontal cortex (PFC) were acquired under fluorescent illumination at 561 nm using a confocal microscope (×63 objective, 1.4 NA) at a pixel resolution of 1024 × 1024 with 2.0 zoom, resulting in a 0.05-µm pixel size. Morphometric analysis of the dendritic spines and calculation of the changes in the spine parameters were performed as described previously (Michaluk et al., 2011; Magnowska et al., 2016; Krzystyniak et al., 2019; Bijata et al., 2022). The images were analyzed semiautomatically using custom software SpineMagick (patent no. WO/2013/021001). The dendritic segments of 3–5 animals were analyzed for each condition; thus, a total of 44–76 dendrite fragments were observed in the Hp in one field of view along the optical axis and 3297–7485 individual dendritic spines were located along the length of the dendrites, for a total length of 3896–6334 μm; in the PFC, a total of 15–20 dendrite fragments were observed in one field of view along the optical axis and 1204–1817 individual dendritic spines were located along the length of the dendrites, for a total length of 864–914 μm. The dendrite fragments in one field of view were used as the statistical unit.

### Analysis of zinc

The samples were mineralized before the experiments. Briefly, three independent test portions of 0.5 g each were weighed from each sample and transferred to Teflon vessels. Then, 2 mL of 30% H_2_O_2_ and 6 mL of 65% HNO_3_ (V) were added, and the mixture was subjected to wet mineralization in a closed system in a Magnum II mineralizer (ERTEC). Mineralization was carried out for 75 min with three cycles of magnetron operation, where the first 30 min was at 60% power, next 15 min was at 80% power, and the remaining 30 min was at 100% power. The mineralized solution thus obtained was heated on a hot plate at 120 °C for 60 min to remove the excess reagents. The samples were then quantitatively transferred to 10 mL flasks and used to determine the zinc content via the F–AAS method (adsorption and emission technique). An atomic absorption spectrometer (iCE 3500, Thermo Scientific, UK) was used to measure the Zn content; each sample was analyzed independently thrice, and the obtained results are presented as mean value ± standard error of mean (SEM).

### Statistical analysis

Statistical analyses were performed using the GraphPad Prism software. Normally distributed data were analyzed by the Student’s t-test and Welch correction in the case of unequal variances (two groups) or one-way ANOVA followed by Newman–Keuls or Sidak’s (multiple groups) *post hoc* tests. Non-normally distributed data were analyzed using the Mann–Whitney test (two groups) or nonparametric Kruskal–Wallis test, followed by Dunn’s *post hoc* test (multiple groups). The statistical significance was set at *p* < 0.05, and no statistical analyses were employed in the pharmacokinetic studies.

## Results

### Effects of Zn-deficient diet on sucrose consumption in the SPT

**FIGURE 1:**
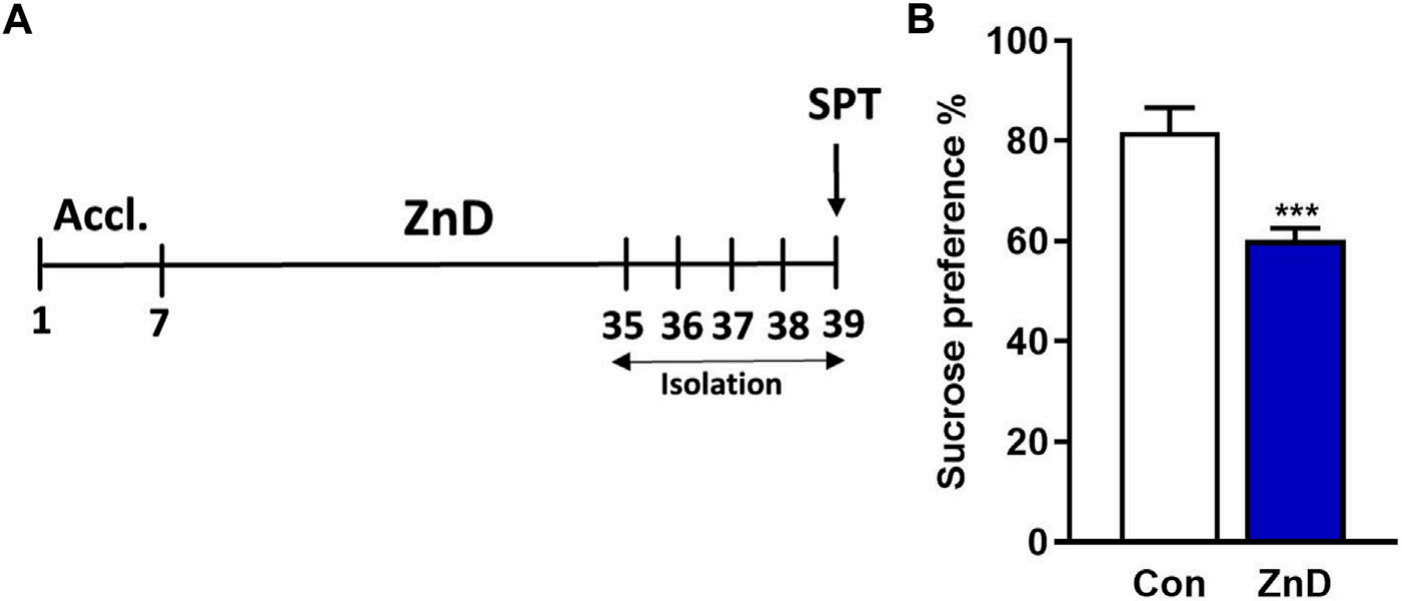
Effects of low-Zn diet in the SPT in mice. **(A)** Experimental schedule. **(B)** SPT. All values are expressed as mean ± SEM. The data were analyzed by Student’s t-test; N = 10, ***p < 0.001.

The results reported from several clinical and preclinical studies suggest a relationship between low Zn levels and depression or depression-like phenotypes (Yang L. et al., 2015; Szewczyk et al., 2018; Antoniuk et al., 2019; Park et al., 2019). Therefore, we intended to determine whether a ZnD could evoke a depression-like phenotype in mice. As seen in Figures 1A,B ZnD induces an anhedonic-like effect that can be observed as a decrease in sucrose preference in mice.

### Effects of Zn-deficient diet on antidepressant-like activity of IMI in the SPT in CRS mice

**FIGURE 2:**
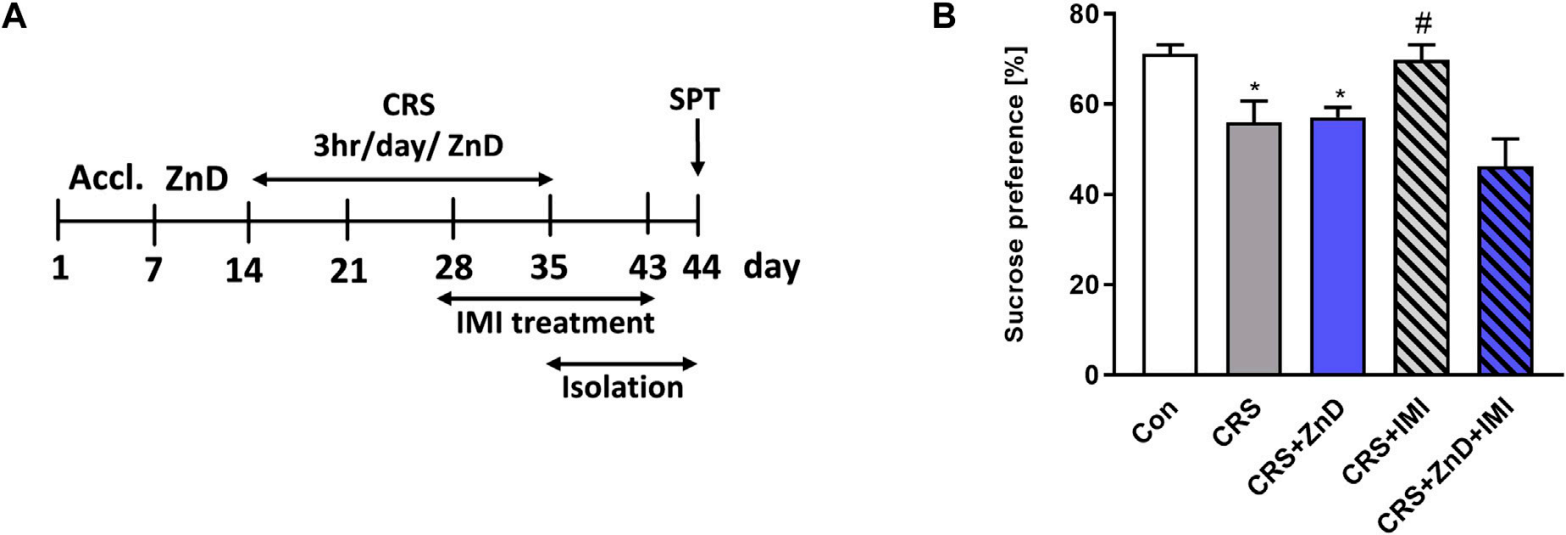
Effects of chronic imipramine treatment in the SPT in mice subjected to CRS and CRS + ZnD. **(A)** Experimental schedule of drug treatments and behavioral tests. **(B)** SPT was performed 24 h after the last treatment. All values are expressed as mean ± SEM. The data were analyzed by one-way ANOVA [F (4, 47) = 7,194; *p* = 0.0001] and Newman–Keuls multiple comparison test, n = 10–11, *p < 0.05 vs. Con; #*p* < 0.05 vs. CRS. Con: control, CRS: chronic restraint stress, ZnD: zinc deficient diet, IMI: imipramine.

Some clinical studies suggest that reduced serum Zn levels can induce refractoriness to classic monoamine-based antidepressants (Maes et al., 1997; Maes et al., 1999; Siwek et al., 2010). Therefore, we wished to determine if the same relationship exists under preclinical conditions. We administered IMI chronically (14 days) to mice subjected to CRS or CRS + ZnD. We chose IMI as a classic reference drug because Zn is known to augment its antidepressant potency under clinical and preclinical conditions (Siwek et al., 2010; Ding et al., 2016; Rafalo-Ulinska et al., 2020). Moreover, its efficacy in CRS mice has been reported previously (Siwek et al., 2010). As shown in Figures 2A,B, chronic administration of IMI restored sucrose preference in CRS mice; however, the same was not observed in CRS + ZnD mice.

### Effects of Zn-deficient diet on antidepressant-like activities of three doses of atypical antidepressant compounds (ketamine, Ro, and Hyp + Lan) in the SPT and TST in CRS mice

**FIGURE 3:**
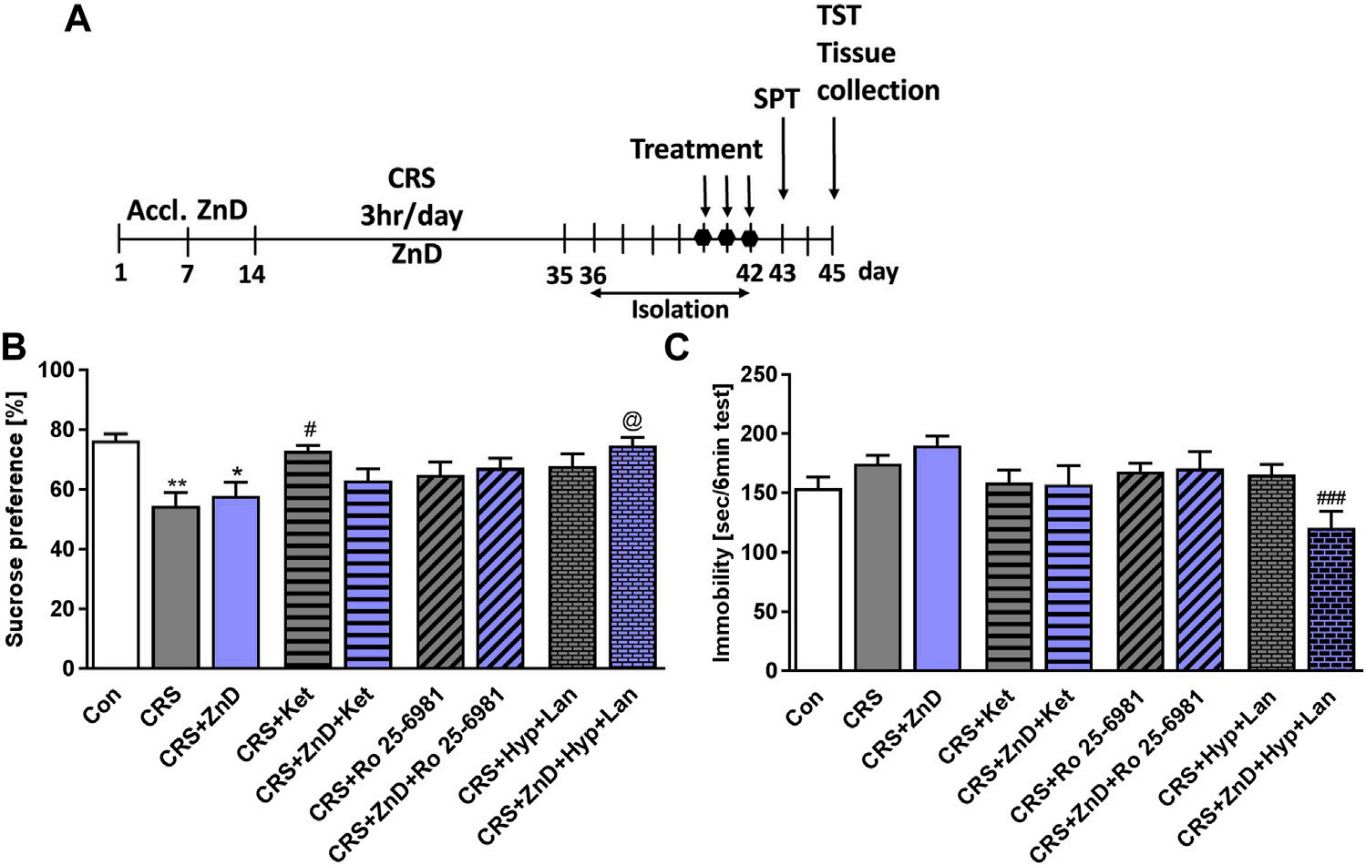
Effects of Zn-deficient diet and CRS on the antidepressant-like activities of three doses of atypical antidepressant compounds (ketamine: 10 mg/kg, Ro 25–6981: 10 mg/kg, Hyp: 2.5 mg/kg + Lan: 10 mg/kg) in the SPT and TST in mice. **(A)** Experimental schedule of drug treatments and behavioral tests. **(B)** SPT was performed 24 h after the last treatment. **(C)** TST was performed 72 h after the last treatment. All values are expressed as mean ± SEM. The data were analyzed by one-way ANOVA and Newman–Keuls multiple comparison test. **(B)** F (8, 92) = 3,948; *p* < 0.0005; *p < 0.05; **p < 0.01 vs. Con, #*p* < 0.05 vs. CRS, ^@^ *p* < 0.05 vs. CRS + ZnD, n = 9–12; **(C)** F (8, 98) = 3,247; *p* = 0.0025; ^###^ *p* < 0.001 vs. CRS + ZnD, n = 10–13. Con: control, ZnD: zinc deficient diet, CRS: chronic restraint stress, Hyp: hyperforin, Lan: lanicemine, SPT: sucrose preference test, TST: tail suspension test.

We previously showed that single doses of ketamine and Ro reversed depression-like behaviors induced by dietary Zn restriction in rats (Pochwat et al., 2020). However, little is known about the efficacies of these compounds in conditions where chronic stress and dietary Zn deficiency coexist. Based on our preliminary studies, we administered three doses of Ro and ketamine in CRS and CRS + ZnD mice. As shown in Figures 3A–C the CRS and CRS + ZnD conditions reduced sucrose preference in mice. Ketamine restored the sucrose preference in CRS animals 24 h after administration. Interestingly, ketamine and Ro both failed to restore sucrose preference in CRS + ZnD mice. Based on our recent finding that a single dose of Hyp + Lan could ameliorate depression-like phenotype in mice chronically treated with corticosterone (Pochwat et al., 2018) and that Hyp regulates intracellular Zn distribution (Tu et al., 2010; Gibon et al., 2011), we investigated whether this drug combination would be effective under CRS + ZnD conditions. We showed that Hyp + Lan was effective in CRS + ZnD mice; however, only a trend was observed in CRS animals (Figure 3B). Seventy-two hours after administering the last doses of the compounds, we performed the TST. No statistical differences were observed between the control and CRS or CRS + ZnD groups with respect to immobility time in the TST (Figure 3C). However, there was a clear trend toward increased immobility time in the CRS + ZnD group compared to the control. Moreover, Hyp + Lan treatment reduced the immobility time for the CRS + ZnD group in the TST (Figure 3C).

### Effects of three doses of atypical antidepressant compounds (ketamine and Hyp + Lan) on the morphologies and densities of dendritic spines in ZnD, CRS, or CRS + ZnD mice

**FIGURE 4:**
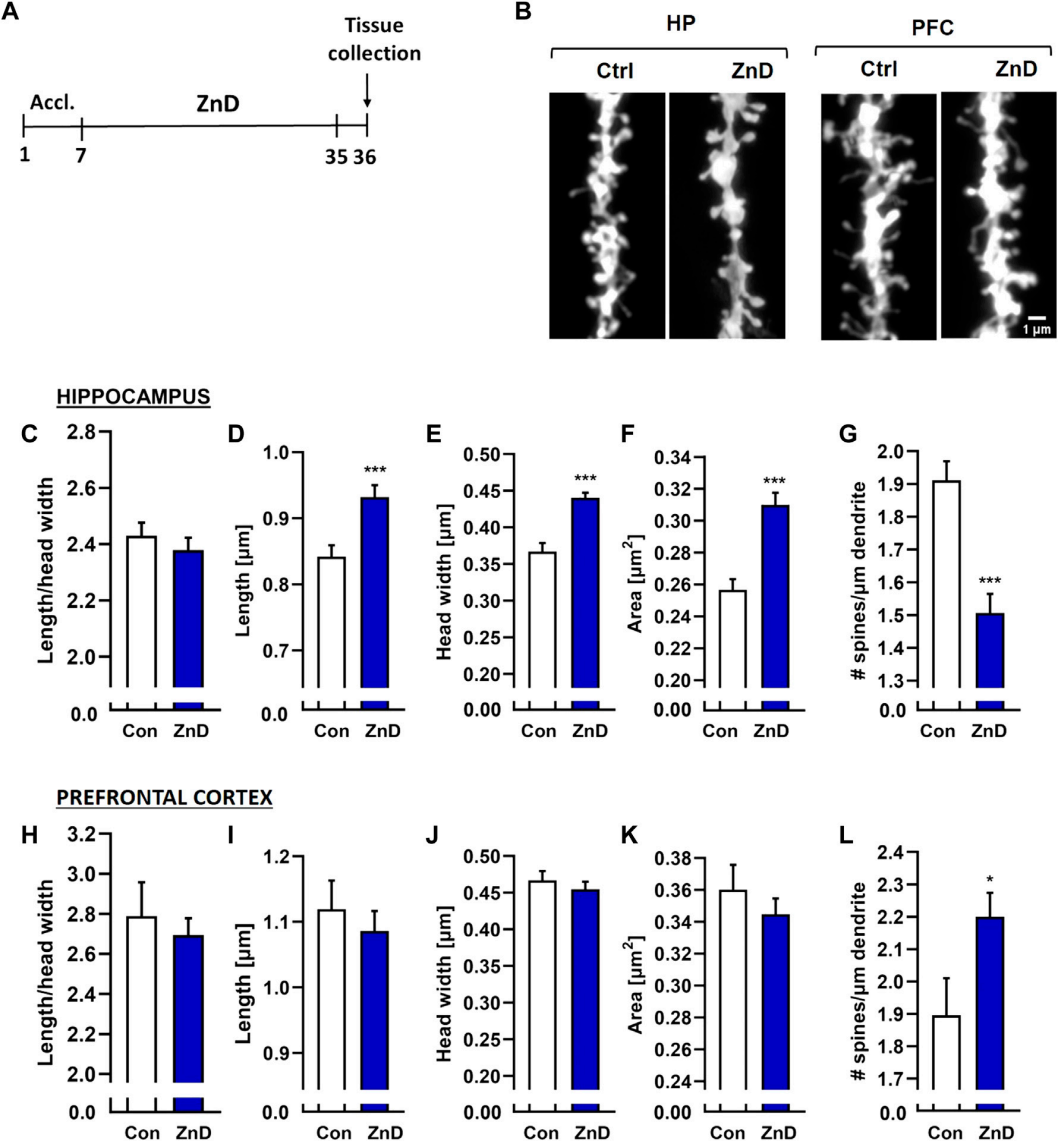
**(A)** Experimental schedule. **(B)** Representative confocal images of DiI-stained dendrite fragments from the hippocampus (Hp) and prefrontal cortex (PFC) of naïve (Con) and zinc-deficient mice. Changes in the shapes and densities of spines in the **(C)** Hp and **(D)** PFC in control and ZnD mice. All values are expressed as mean ± SEM. The data were analyzed by Mann–Whitney test [Hp, **(C–G)**], Student’s t-test with Welch’s correction [PFC, **(H)**], and Student’s t-test [FC, **(I–L)**]. [ZnA: **morphology**: n mice = 4, n dendrites = 66, n spines = 7845; **density**: n mice = 4, dendrites = 65, total dendrite length = 6334 μm; ZnD: **morphology**: n mice = 4, n dendrites = 55, n spines = 4489; **density**: n mice = 4, n dendrites = 57, total dendrite length = 4202 μm]; FC [ZnA: **morphology**: n mice = 4, n dendrites = 15, n spines = 1204; total dendrite length = 864 μm; ZnD: **morphology**: n mice = 4, n dendrites = 19, n spines = 1817; **density**: n mice = 4, dendrites = 20, total dendrite length = 914 μm]. *p < 0.05, ***p < 0.001 vs Con. Con: control, ZnD: zinc defcient diet.

**FIGURE 5:**
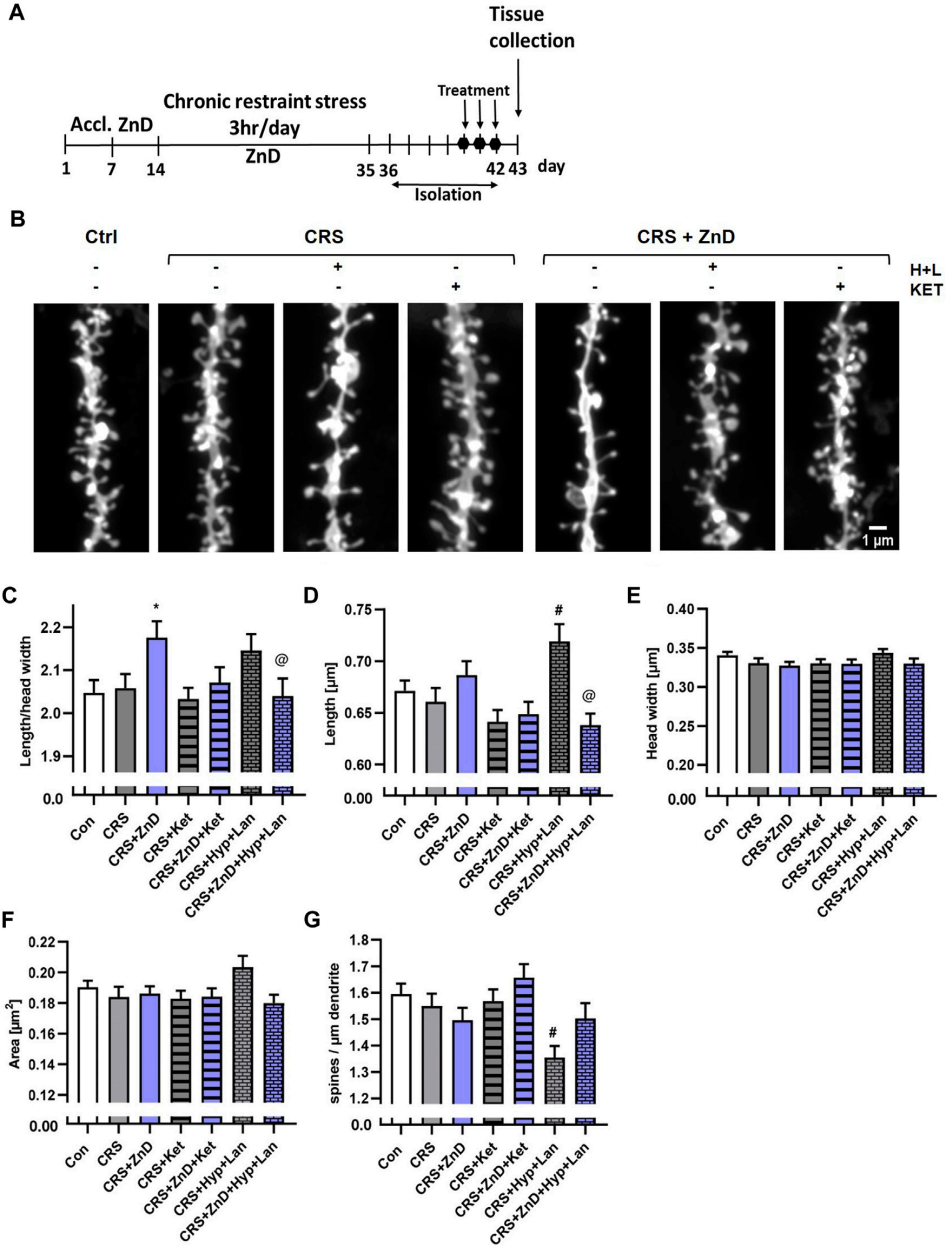
Effects of three doses of atypical antidepressant compounds (ketamine and Hyp + Lan) on the morphologies and densities of dendritic spines in the Hp in mice subjected to CRS, or CRS + ZnD. **(A)** Experimental schedule. **(B)** Representative confocal images of DiI-stained dendrite fragments. Changes in the shape and density of spines in the **(C)** Hp of mice. All values are expressed as mean ± SEM. The data were analyzed by **(C–F)** Kruskal–Wallis test followed by Dunn’s multiple comparison test or **(G)** one-way ANOVA followed by Sidak’s multiple comparison test. * *p* < 0.05 vs Con, @ *p* < 0.05 vs CRS + ZnD, # *p* < 0.05 vs CRS. Hp: [**morphology**: Con: n mice = 5, n dendrites = 76, n spines = 6286; CRS: n mice = 5, n dendrites = 60, n spines = 4760; CRS + Hyp + Lan: n mice = 4, n dendrites = 44, n spines = 3297; CRS + Ket: n mice = 4, n dendrites = 67, n spines = 5064; CRS + ZnD: n mice = 4, n dendrites = 59, n spines = 4427; CRS + ZnD + Hyp + Lan: n mice = 4, n dendrites = 61, n spines = 4863; CRS + ZnD + Ket: n mice = 3, n dendrites = 60, n spines = 4937; **density**: Con: n mice = 5, n dendrites = 76, total dendrite length = 5009 μm; CRS: n mice = 5, n dendrites = 60, total dendrite length = 3896 μm; CRS + Hyp + Lan: n mice = 4, n dendrites = 44, total dendrite length = 3010 μm; CRS + Ket: n mice = 4, n dendrites = 67, total dendrite length = 4651 μm; CRS + ZnD: n mice = 4, n dendrites = 59, total dendrite length = 3908 μm; CRS + ZnD + Hyp + Lan: n mice = 4, n dendrites = 59, total dendrite length = 4054 μm; CRS + ZnD + Ket: n mice = 3, n dendrites = 60, total dendrite length = 4040 μm]. Con: control, ZnD: zinc deficient diet, Ket: ketamine, Hyp: hyperforin, Lan: lanicemine.

The decreased dendritic spine densities in rodents subjected to different paradigms of chronic stress are well known (Li et al., 2011; Magarinos et al., 2011; Qiao et al., 2016; Ng et al., 2018; Zhang et al., 2019; Ma et al., 2021). Alterations in the dendritic spine densities have been reported in a few brain structures, including the Hp, FC, and amygdala (Li et al., 2011; Magarinos et al., 2011; Qiao et al., 2016; Ng et al., 2018; Zhang et al., 2019; Ma et al., 2021). Furthermore, reductions in Zn levels in the brain have been known to alter dendritic spine densities in rodents. Zinc deficiency induced by a two-week administration of the Zn (and Cu) chelator 5-chloro-7-iodo-8-hydroxyquinoline (clioquinol) reduced the dendritic spine density in the CA1 region of the mice Hp (Frazzini et al., 2018). Conversely, rats fed a Zn-deficient diet for 4 weeks exhibited increased dendritic spine densities in the cingulate and infralimbic cortexes (Pochwat et al., 2020). Interestingly, fast-acting and long-lasting antidepressant compounds, such as ketamine or its enantiomers, and recently psilocybin alter the density of the dendritic spines (Li et al., 2011; Yang C. et al., 2015; Ng et al., 2018; Zhang et al., 2019; Shao et al., 2021) or their morphology (Ma et al., 2021; Shao et al., 2021) in chronically stressed animals. Moreover, chronic administration of Hyp improved the cognitive abilities in rats subjected to chronic unpredictable stress, increasing their dendritic spines densities (Liu et al., 2015). We investigated whether dietary Zn restriction (Figures 4A–L) and CRS + ZnD (Figures 5A–G) could alter the dendritic spine density and/or morphology. We also explored how these potential alterations are modified by atypical antidepressant compounds (ketamine and Hyp + Lan). Figure 4L shows that the dendritic spine density increased in the PFC after 4 weeks of dietary Zn restriction. In contrast, there were more pronounced effects of ZnD in the Hp (Figures 4C–G). Mice fed a modified fodder showed decreased dendritic spine densities in the Hp compared to control mice (Figure 4G). Moreover, morphometric analysis indicated that the dendritic spines in the Hp of the ZnD mice had increased length, head width, and area (Figures 4C–F). Since the dietary Zn restriction induced more pronounced changes in the Hp, the subsequent studies were conducted using hippocampal tissue. The combination of CRS and ZnD did not induce statistically significant changes in the following morphometric parameters: spine length, spine width, area, and density (Figures 5D–G). However, the length/head width ratio was higher in the CRS + ZnD group compared to the control group (Figure 5C). These alterations were reversed by three doses of Hyp + Lan but not ketamine (Figure 5C). Additionally, morphometric analysis showed reduced density and spine elongation after Hyp + Lan treatment in the CRS group compared to the CRS + ZnD mice (Figure 5G).

### Effects of three doses of atypical antidepressant compounds (ketamine, Ro, and Hyp + Lan) on Zn levels in the Hp and FC of CRS and CRS + ZnD mice

**FIGURE 6:**
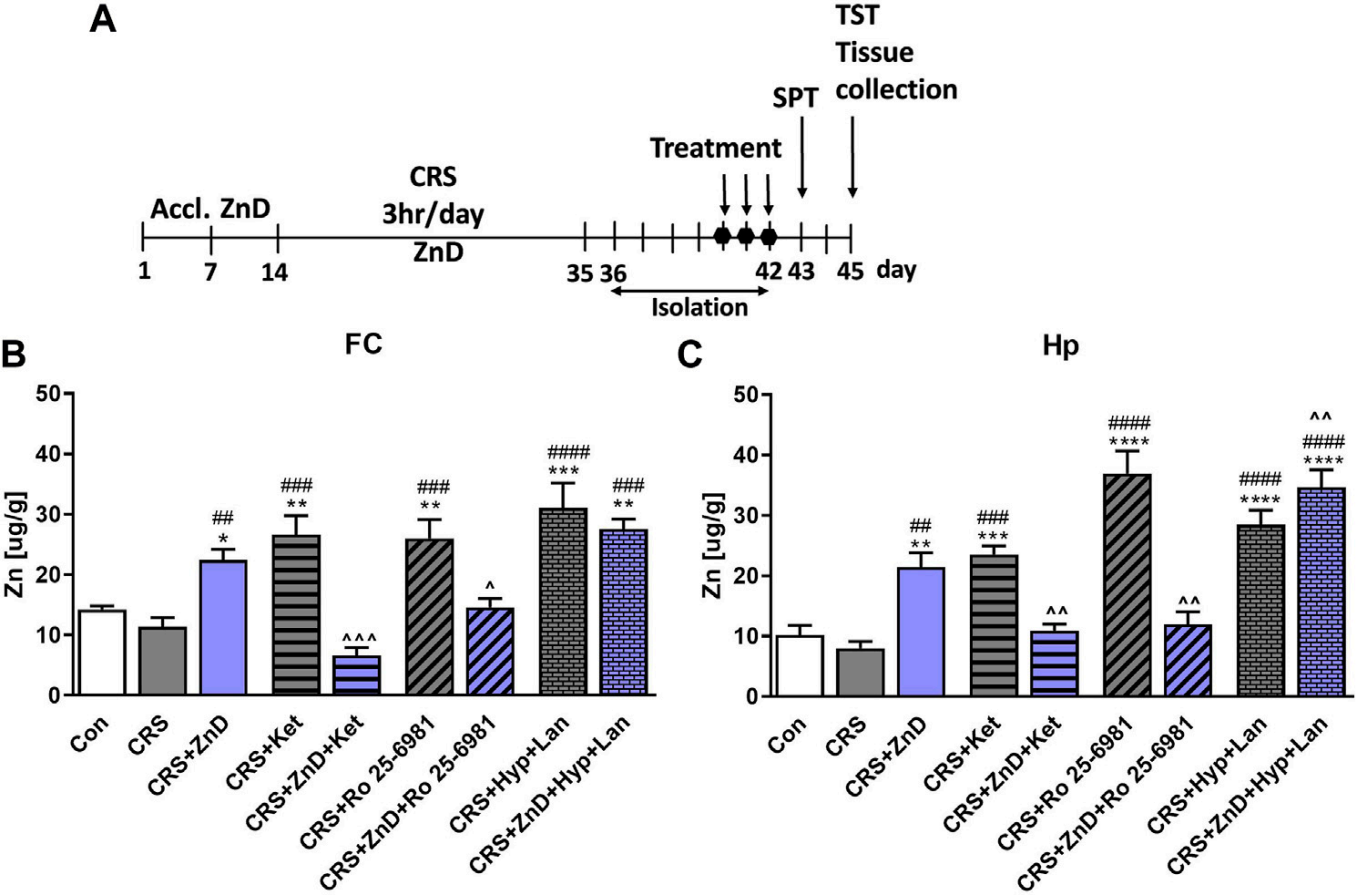
Effects of three doses of atypical antidepressant compounds (ketamine: 10 mg/kg, Ro 25–6981: 10 mg/kg; Hyp: 2.5 mg/kg + Lan: 10 mg/kg) on Zn levels in the FC and Hp of mice subjected to CRS and CRS + ZnD. **(A)** Experimental schedule of drug treatments and behavioral tests. **(B)** Zn level in the FC and **(C)** Zn level in the Hp. All values are expressed as mean ± SEM, n = 5–6. The data were analyzed by one-way ANOVA and Newman–Keuls multiple comparison test. FC: [F(8.45) = 13,15; *p* < 0.0001]; Hp: [F(8.42) = 23.56; *p* < 0.0001] *p < 0.05, **p < 0.01, ***p < 0.001, ****p < 0.0001 vs. Con; ^##^ *p* < 0.01, ^###^ *p* < 0.001, ^####^ *p* < 0.0001 vs. CRS, ^*p* < 0.05, ^ ^*p* < 0.01, ^ ^ ^*p* < 0.001 vs. CRS + ZnD. Con: control, CRS: chronic restraint stress, Hyp: hyperforin, Ket: ketamine, Lan: lanicemine.

It has been reported previously that dietary Zn restriction (4 weeks) lowers the Zn levels in the PFC and Hp of rats (Doboszewska et al., 2016). However, 1 or 2 weeks of psychological stress reduced the hippocampal Zn levels in rats (Dou et al., 2014). We investigated the relationships between these factors because little is known about the combined effects of dietary Zn restriction and CRS on the cortical and total hippocampal Zn levels (Figures 6A–C). Furthermore, we wanted to determine if the antidepressant-like effects of atypical compounds correlated with the Zn levels. Surprisingly, dietary Zn restriction in CRS mice enhanced the Zn levels in the Hp and FC compared to the control and CRS groups (Figures 6B,C). Interestingly, Ro and ketamine, which did not evoke antidepressant effects in these mice, reduced the Zn levels in the Hp and FC (Figures 6B,C). Conversely, active Hyp + Lan increased the Zn levels in the FC and Hp in the CRS + ZnD group (Figures 6B,C). There were no significant changes in the Zn levels in the CRS animals compared to the controls. However, all drugs administered to the CRS group increased the Zn levels compared to the controls (Figures 6B,C).

### Metabolism and distribution of ketamine in CRS and CRS + ZnD mice

**FIGURE 7:**
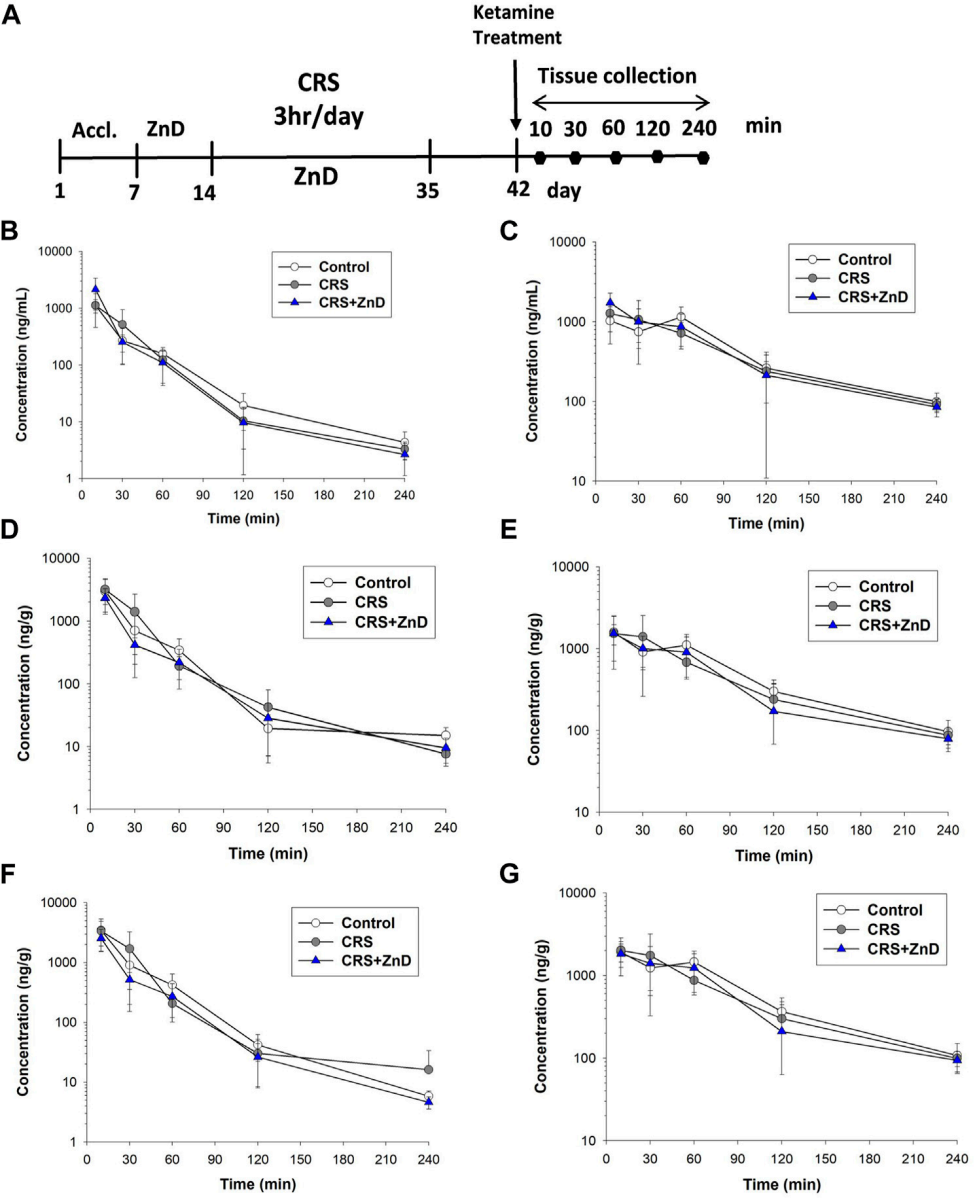
**(A)** Experimental schedule of drug treatment. **(B,C)** Concentration vs. time profiles of ketamine (left panel) and its metabolite hydroxynorketamine (right panel) in mouse serum following intraperitoneal (i.p.) administration of 10 mg/kg ketamine (n = 6). **(D,E)** Concentration vs. time profiles of ketamine (left panel) and its metabolite hydroxynorketamine (right panel) in mouse hippocampus following i.p. administration of 10 mg/kg ketamine (n = 6). **(F,G)** Concentration vs. time profiles of ketamine (left panel) and its metabolite hydroxynorketamine (right panel) in mouse frontal cortex following i.p. administration of 10 mg/kg ketamine (n = 6). CRS: chronic restraint stress, ZnD: zinc deficient diet.

**FIGURE 8:**
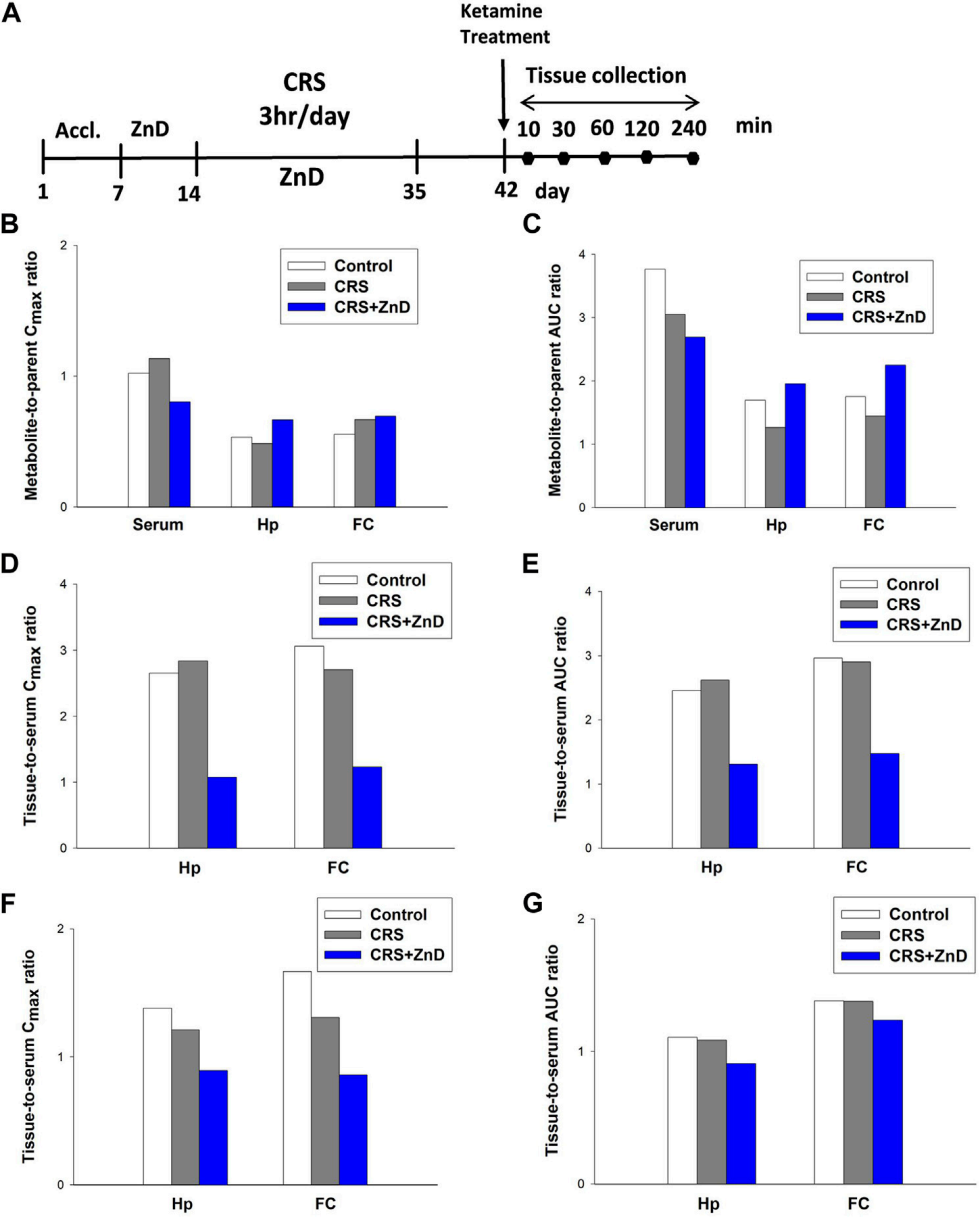
**(A)** Experimental schedule of drug treatment. **(B,C)** Metabolite-to-parent ratios of maximum concentrations (C_max_; left panel) and areas under the curves (AUCs; right panel) in the control and study groups. **(D,E)** Tissue-to-serum ratios of C_max_ (left panel) and AUCs (right panel) for ketamine in the control and study groups. **(F,G)** Tissue-to-serum C_max_ (left panel) and AUCs (right panel) for (2R,6R;2S,6S)-HNK in the control and study groups. CRS: chronic restraint stress, FC: frontal cortex, Hp: hippocampus, ZnD: zinc deficient diet.

As described previously, ketamine did not reverse the behavioral disturbances observed in CRS + ZnD animals but was effective in CRS animals. Recently, several studies have elaborated on the significance of the metabolism of ketamine and its antidepressant efficacy in preclinical conditions, emphasizing the role of (2R,6R)-hydroxynorketamine (2R,6R-HNK) in the ketamine-dependent antidepressive effects (Zanos et al., 2016; Zanos et al., 2018). Ketamine is initially metabolized to norketamines and converted to (2R,6R;2S,6S)-hydroxynorketamine (2R,6R;2S,6S)-HNK) by two isoforms of the hepatic enzyme CYP 450 (CYP2A6 and CYP2B6) (Zanos et al., 2018). It is notable that chronic Zn depletion in rats reduced the rates of metabolism of some compounds, such as pentobarbital, aminopyrine, or p-nitrobenzoic acid (Becking and Morrison, 1970). Based on the above assumptions, we conducted pharmacokinetic studies of ketamine in the control, CRS, and CRS + ZnD mice. As shown in Figures 7A–G and Table 1, concentrations of both analytes were measurable for up to 240 min in the serum and brain structures tested. Both ketamine and its metabolite reached peak concentrations very quickly in the serum (Figures 7B, C) and studied brain structures (t_max_ = 10 min) in all mice groups, except for (2R,6R;2S,6S)-HNK in the control group, where the time to reach maximum concentration was 60 min. This indicates that CRS + ZnD may increase ketamine first-pass metabolism in the liver. In the CRS + ZnD group, this effect was further augmented by the increased absorption of ketamine from the peritoneal cavity as ketamine C_max_ in this experimental group was almost twice as high as those in the other groups under investigation (2148.83 vs. 1127.83 and 1118.33 ng/mL, Table 1). Moreover, both factors did not influence its elimination from the blood as comparable terminal slopes (λ_z_) elimination half-lives, and MRTs were observed in all animal groups. The (2R,6R;2S,6S)-HNK reached relatively high concentrations in all tissues analyzed. The metabolite-to-parent C_max_ ratios in serum were similar for both control and study groups and were close to 1, whereas they were about half of the observed serum values in the studied brain structures (Figures 8A–C). Interestingly, in contrast to the serum, these ratios tended to be higher in the Hp and FC in the CRS + ZnD group compared to the control animals (Figures 8B,C). This indicates that Zn deficiency may influence (2R,6R;2S,6S)-HNK pharmacokinetics in these brain regions following ketamine administration. Comparing the tissue-to-serum C_max_ and AUC ratios for the parent drug (Figures 8D,E), the values were similar for both the control and stress groups (2.45–2.84), whereas they were much lower in the CRS + ZnD animals (C_max_ =1 and AUC =1.3). As shown in Figures 8F,G, the ratios for the metabolites showed the same patterns; however, they were about 40% of the values observed for the parent compound in the serum and Hp and about 80% in the FC. Consequently, the differences between the CRS + ZnD and other groups were less evident than those in the case of the parent drug.

**TABLE 1:** Pharmacokinetic parameters of ketamine and hydroxynorketamine in mouse serum and brain structures estimated using noncompartmental analysis following administration of the parent drug at an i.p. dose of 10 mg/kg in the control and study groups.

Parameter	Control and treatment groups					
	Serum					
	Ketamine			Hydroxynorketamine		
	Control	CRS	CRS + ZnD	Control	CRS	CRS + ZnD
t_max_ (min)	10	10	10	60	10	10
C_max_ (ng/mL)	1127.83	1118.33	2148.83	1152.71	1269.00	1726.00
λ_z_ (min^−1^)	0.019	0.019	0.019	0.011	0.012	0.013
t_0.5_λ_z_ (min)	36.70	37.06	35.93	62.33	59.11	56.28
AUC_0-∞_ (µg·min/L)	33.00	36.70	44.66	124.21	111.99	120.29
V_z_/F (L/kg)	16.04	14.57	11.61	—	—	—
CL/F (L/min/kg)	0.30	0.27	0.22	—	—	—
MRT (min)	34.17	29.83	22.00	87.69	81.05	73.20

	Hippocampus					
	Ketamine			Hydroxynorketamine		
	Control	CRS	CRS + ZnD	Control	CRS	CRS + ZnD
t_max_ (min)	10	10	10	10	10	10
C_max_ (ng/g)	2990.00	3176.67	2313.33	1591.33	1536.83	1540.00
λ_z_ (min^−1^)	0.019	0.017	0.016	0.012	0.013	0.013
t_0.5_λ_z_ (min)	37.36	39.68	42.69	58.88	54.52	54.06
AUC_0-∞_ (µg·min/g)	80.90	96.13	58.49	137.36	121.64	114.29
MRT (min)	30.39	28.48	31.33	80.93	72.78	70.35

	Frontal cortex					
	Ketamine			Hydroxynorketamine		
	Control	CRS	CRS + ZnD	Control	CRS	CRS + ZnD
t_max_ (min)	10	10	10	10	10	10
C_max_ (ng/g)	3455.00	3028.00	2649.00	1920.83	2020.00	1838.33
λ_z_ (min^−1^)	0.023	0.020	0.022	0.014	0.012	0.013
t_0.5_λ_z_ (min)	30.49	34.92	32.31	50.01	59.38	52.49
AUC_0-∞_ (µg·min/g)	97.81	106.58	65.97	171.66	154.18	148.57
MRT (min)	30.05	28.44	28.79	74.68	72.72	68.20

From Table 1, it is seen that elimination of (2R,6R;2S,6S)-HNK from the murine serum and brain structures was slower than that of the parent drug, as reflected by a nearly two-fold higher terminal half-life and MRT of this metabolite in these tissues. These values were similar in all animal groups studied, indicating that the experimental protocol did not influence the elimination of ketamine and its metabolite from the mouse body. The lower value of CL/F observed in the CRS + ZnD group than the other animal groups studied is probably a result of the higher bioavailability (F) of ketamine after i.p. administration in this group. The same reasoning applies to the lower value of V_z_/F estimated in the CRS + ZnD group. Thus, a pharmacokinetic study after intravenous administration of ketamine is necessary to confirm this assumption.

### Effects of CRS and CRS + ZnD on mice body weights

Table 2 shows that mice subjected to CRS and CRS + ZnD had significantly lower weight gains than the control group. There were no differences between the CRS and CRS + ZnD groups.

**TABLE 2:** Influences of CRS and CRS + ZnD on mice weight.

	Δ Weight [g] ± SEM
Con	3.608 ± 0.267
CRS	0.678 ± 0.239^a^
CRS + ZnD	0.488 ± 0.208^a^

^a^Data were analyzed using one-way ANOVA [F (2, 92) = 27,11, *p* < 0.0001] and Newman–Keuls *post hoc* test *p* < 0.0001 vs. Con. (n = 13–41). Weights were determined from days 14 to 42 of the experiments (see Figure 7A).

## Discussion

Clinical and preclinical data have shown that dietary Zn deficiency may be associated with the development of MDD in humans, resistance to monoamine-based antidepressant drugs, and expression of depression-like phenotype in laboratory animals (Hansen et al., 1983; Maes et al., 1997; Marcellini et al., 2006; Jacka et al., 2012; Lehto et al., 2013; Siwek et al., 2013; Vashum et al., 2014; Markiewicz-Zukowska et al., 2015). Numerous studies devoted to the etiology of MDD suggest that the biological and environmental bases of MDD are very complex and dependent on many factors. Among them, chronic stress or dietary habits and their precise roles in MDD development are still under debate (Yang L. et al., 2015; Szewczyk et al., 2018; Park et al., 2019; Cruz-Pereira et al., 2020). Based on the aforementioned premise, we investigated two main questions. First, can low dietary Zn and CRS in mice induce resistance to atypical antidepressant compounds, such as ketamine, Ro, and Hyp + Lan? Second, how do dietary Zn levels affect the efficacies of classic monoamine drugs in CRS? Finally, we wanted to determine the possible biological events associated with resistance to the atypical compounds used in this study.

Our first experiment showed that ZnD alone induced anhedonia-like effects in the SPT in mice. Next, we showed that 2 weeks of IMI (10 mg/kg) treatment reversed CRS-induced anhedonia in SPT in mice. We thus confirmed the previously reported antidepressant efficacy of IMI in CRS in the TST (Ding et al., 2016) or social interaction test (Leem et al., 2020). In contrast, ZnD induced resistance to IMI in CRS mice in the SPT, akin to clinical conditions in which patients resistant to IMI exhibited lower serum Zn levels than IMI-treated nonresistant patients (Siwek et al., 2010).

Because several clinical studies have reported the efficacy of ketamine in treatment-resistant depression (Pochwat et al., 2019), we sought to determine if a combination of CRS and ZnD produced a ketamine-resistant phenotype in mice. We showed that ketamine administered consecutively for 3 days exhibited antidepressant-like activity in the SPT in CRS mice 24 h after the last dose, confirming its widely reported antidepressant potency in stress-related paradigms in preclinical studies (Pochwat et al., 2019). The other atypical compounds (Hyp + Lan and Ro) did not show any statistically significant antidepressant-like activities in the SPT. However, there was a trend toward antidepressant-like activity in the Hyp + Lan group. Moreover, we found no statistically significant differences between the control and stress groups or CRS + ZnD group in the TST 3 days after the last dose of each atypical compound and 7 days after stress cessation. However, Hyp + Lan decreased the immobility time in the CRS + ZnD group.

Because the reduced dietary Zn levels induced resistance to pharmacological treatment, we wanted to determine whether the antidepressant-like activities of atypical drugs somehow correlated with the Zn concentrations in the Hp and FC, which are two crucial brain structures involved in the regulation of depression-like behaviors. We also checked the effects of CRS and CRS + ZnD with respect to this parameter. Contrary to our previously published studies on the relationships between stress and hippocampal as well as cortical Zn levels (Szewczyk et al., 2019), we found no changes in the Zn levels in CRS animals. However, these discrepancies can be explained by the different types of stresses utilized (CRS vs. chronic unpredictable stress) and differences in the duration of stress (3 vs. 7 weeks) (Szewczyk et al., 2019). Interestingly, all the atypical compounds enhanced the total Zn levels in the FC and Hp of the stressed animals. However, the increases in hippocampal and cortical Zn levels correlated only with the antidepressant-like effects of ketamine. One fascinating revelation is that ketamine and Ro, while ineffective in the SPT and TST, decreased the hippocampal and cortical Zn levels in the CRS + ZnD group compared to the CRS + ZnD vehicle-treated animals. Surprisingly, CRS + ZnD mice had increased cortical and hippocampal Zn levels than the control animals.

It is challenging to explain this observation at present. However, some studies show that Zn deficiency could be an important factor in the loss of integrity of the blood-brain barrier under pathological conditions, such as hyperoxia (Noseworthy and Bray, 2000). This disintegration is dependent on oxidative stress measured through the ratio of oxidized to reduced glutathione (Noseworthy and Bray, 2000). In parallel, some studies have indicated that CRS in mice could induce disturbances in the glutathione-dependent intracellular processes (Ejchel-Cohen et al., 2006). This case is more complex because the Zn levels in the brain are also regulated by Zn-specific transporters, which may be dysregulated by stress, resulting in abnormal blood Zn levels (Tian et al., 2014). Therefore, it is likely that the interactions between chronic stress and low dietary Zn levels can evoke paradoxical changes in the Zn levels in the brain by disrupting the blood-brain barrier integrity and levels or activity of Zn transporters regulating Zn homeostasis in the brain. Zn redistribution between the regions of the brain is another plausible explanation for the changes in the brain Zn levels induced by the combination of CRS and ZnD; this may explain why all the atypical drugs enhanced hippocampal and cortical Zn levels in the stress groups or why the Hyp + Lan enhanced the Zn levels in the CRS + ZnD group. This manner of Zn redistribution has been reported after chronic IMI treatment and electroconvulsive shock in rats (Nowak and Schlegel-Zawadzka, 1999). For now, we can only speculate on the mechanism responsible for the changes in the brain Zn levels until further studies can be conducted.

The next fascinating phenomenon is the lack of efficacies of ketamine and Ro in CRS + ZnD mice and the effectiveness of Hyp + Lan treatment. The intriguing thing here is that the administration of ketamine and Ro induced completely different effects on the cortical and hippocampal Zn levels than Hyp + Lan. Three doses of ketamine or Ro reduced the Zn levels in the FC and Hp of CRS + ZnD mice. In contrast, we observed increased Zn levels in both brain structures after Hyp + Lan treatment than the control group. Considering that the antidepressant-like activities of ketamine in CRS and Hyp + Lan in CRS + ZnD are associated with elevated brain Zn levels, it is suggested that the lack of increases in Zn levels in the cases of ketamine and Ro contributes to their lack of antidepressant potency. The antidepressant mechanisms of N-methyl-D-aspartate receptor (NMDAR) antagonists are very complex; it is postulated that these compounds block different types of NMDARs that are differentially localized in the neurons, such as synaptic vs. extrasynaptic and GABA-interneurons vs. glutamate pyramidal neurons (Pochwat et al., 2019). Possibly, all of these synaptic events may be important for complete antidepressant response. The biological effects induced by NMDAR antagonists, such as ketamine or Ro, also depend on enhanced glutamate release or cycling in the FC or Hp (Chowdhury et al., 2017; Widman and McMahon, 2018). In parallel, it is well known that some pyramidal glutamate neurons found in the Hp or cortex are zincergic (Frederickson, 1989; Brown and Dyck, 2004); in other words, they contain Zn stored in synaptic vesicles, which are released in a Ca^2+^-dependent manner. Zn released from these neurons block the postsynaptic NMDARs (Howell et al., 1984; Amico-Ruvio et al., 2011). Moreover, recent studies have suggested that Zn can be released into the synaptic cleft by postsynaptically localized ZnT1 transporters (Krall et al., 2020). Therefore, the increased Zn levels observed in the CRS + ketamine as well as CRS + ZnD and Hyp + Lan groups may support the blockade of the NMDARs, which is crucial for the antidepressant-like activities of these substances.

The next question raised by the present results is why only Hyp + Lan evoked increase in the Zn levels in CRS + ZnD mice. This phenomenon is probably dependent on the biological activity of Hyp. The antidepressant-like activity of Hyp has been described in several studies (Chatterjee et al., 1998; Pochwat et al., 2018; Szewczyk et al., 2019). However, its precise molecular mechanism of action is unknown; it has a multimodal mechanism of action involving several molecular targets. In the present context, *in vitro* studies showed that Hyp can alter the Zn storage capacities of brain cells. Chronic Hyp treatment either *in vitro* (cortical neurons) or *in vivo* (in mice) induces enhanced levels of metallothioneins, which are responsible for Zn storage (Gibon et al., 2011), thereby retaining Zn in the brain. However, further studies are required to understand whether Hyp + Lan has any effect on metallothioneins in the CRS + ZnD mice as a potential mechanism that allows the Hyp + Lan-treated group to sequester Zn in the Hp and FC.

Since ketamine was not effective in the CRS + ZnD mice, we wanted to evaluate whether CRS or CRS + ZnD could alter the metabolism or bioavailability of ketamine. Preclinical studies have shown that the antidepressant effects of ketamine are strongly linked to the activity of 2R,6R*-*HNK (Zanos et al., 2016). It is worth noting that Zn deficiency could change the metabolism of certain drugs in rodents (Becking and Morrison, 1970). However, it is unknown if the same is true for ketamine. Our pharmacokinetic results for CRS and CRS + ZnD showed no alterations in the liver metabolism of ketamine as the serum levels of (2R,6R;2S,6S)-HNK were similar for all groups. Thus, altered metabolism of ketamine does not account for the lack of its antidepressant-like activity in the CRS + ZnD group. However, comparing the metabolite-to-parent AUC ratios in the Hp and FC of the CRS + ZnD group to the controls, the values tended to be higher, suggesting different sources of influence for HNK pharmacokinetics in these brain structures in the CRS + ZnD mice. Although the liver metabolism of ketamine to (2R,6R;2S,6S)-HNK is not altered in the CRS + ZnD group, the bioavailability of the parent drug is entirely different than that of the control group. CRS + ZnD mice had two-fold higher serum ketamine levels. More importantly, the enhanced serum ketamine levels did not mirror the brain concentrations of the drug. We can thus see that CRS + ZnD mice have much lower hippocampal and cortical ketamine levels than the control or CRS groups. The same pattern was observed for (2R,6R;2S,6S)-HNK, albeit with lower differences. The lower brain levels of ketamine and its metabolite may be a potential reason why ketamine is inactive in the CRS + ZnD mice. Many factors could cause discrepancies in the ketamine levels among the CRS + ZnD, CRS, and control groups. For instance, ketamine is a substrate of P-glycoprotein and breast cancer resistance proteins that are responsible for drug efflux from the brain and regulation of drug concentrations in the serum and other tissues (Ganguly et al., 2018). Moreover, some studies have shown that *R-*ketamine could alter the blood-brain barrier integrity under some conditions (Choudhury et al., 2021). Therefore, tissue and brain concentrations of ketamine could be functions of at least two factors, such as expression of the drug efflux proteins and integrity of the blood-brain barrier, which can be perturbed by alterations in the Zn levels.

In the last series of experiments, we evaluated the effects of ZnD, CRS, CRS + ZnD, and atypical drugs on the morphology and density of hippocampal dendritic spines. The harmful effects of different chronic stress paradigms on hippocampal dendritic spine density and morphology in mice have been reported previously (Yang C. et al., 2015; Ng et al., 2018; Zhang et al., 2019). CRS is known to reduce dendritic spine density and lead to atrophy in the CA1 region of the Hp (Magarinos et al., 2011). Zn deficiency induced by chronic clioquinol-Zn (Cu) chelator treatment has been shown to reduce spine density in the mouse Hp (Frazzini et al., 2018). Moreover, the potency to enhance dendritic spine density and influence dendritic spine morphology are postulated as the primary cellular effects of ketamine and other fast-acting antidepressants (Yang C. et al., 2015; Ng et al., 2018; Zhang et al., 2019; Shao et al., 2021). Similar to results obtained in our previous studies in rats (Pochwat et al., 2020), dietary Zn restriction increased the dendritic spine density in the FC; in the Hp, we noted the opposite effect. These results are in accordance with reported studies showing the effects of clioquinol on dendritic spine density in the hippocampal CA1 region (Frazzini et al., 2018). Dietary Zn restriction significantly alters the morphology of dendritic spines, resulting in spines with longer necks and increased head areas. The physiological significance of these alterations is rather speculative and requires further studying. Contrary to other published works (Magarinos et al., 2011), CRS did not alter the density and morphology of the hippocampal dendritic spines; the influences of ketamine and Hyp + Lan on these parameters are also very limited. In other words, the behavioral disturbances observed in the CRS group and antidepressant-like effects of ketamine cannot simply be explained by the altered density or morphology of the hippocampal dendritic spines. Discrepancies between our results and those published previously can be explained by the difference in the CRS duration (3 h in our studies vs. 6 h in others) or time points of tissue collection (7 days after stress cessation in the present study vs. 1 day in others). It is, therefore, possible that we may have observed some overlapping effects of stress and recovery from stress, resulting in the differences between our work and other previously reported studies. It should be noted, however, that 72 h after the last dose of ketamine and 7 days after stress cessation, we did not observe any behavioral effects of CRS in the TST. In contrast, the CRS + ZnD group had spines with increased length/head width ratio in the Hp compared to the control group. This parameter describes the maturity of the dendritic spines and suggests that the dendritic spines are remodeled to less mature structures characterized by thin and narrow spines with relatively small heads (Duman and Duman, 2015; Bijata et al., 2017; Baczynska et al., 2021; Bijata et al., 2022). Synapses formed by less mature spines are weaker and may not process information effectively, resulting in a depression-like phenotype. Interestingly, the administration of Hyp + Lan reversed these disturbances in the CRS + ZnD group. Because ketamine and Lan are both NMDAR antagonists and Hyp + Lan was effective in the above experimental group, several explanations of this phenomenon can be considered. First, based on our pharmacokinetic results, the concentration of ketamine could be too low to restore dendritic spine morphology. Second, a possible solution may be associated with low brain Zn levels observed after ketamine administration. Third, a potential mechanism of action of Hyp may be at play. As mentioned earlier, the exact antidepressant mechanism of Hyp is not fully understood; however, a few *in vivo* and *in vitro* studies indicate TRPC6 receptor stimulation as the focal point for the molecular action of Hyp (Tu et al., 2010; Gibon et al., 2013; Liu et al., 2015; Pochwat et al., 2018). The functional relationship between TRPC6 receptor stimulation and effects of Hyp on intracellular Zn pools should be considered. Furthermore, *in vitro* studies show that Hyp increases the ratio of mature stubby spines in the CA1 and CA3 pyramidal neurons and reduces the proportion of thin spines in the CA1 pyramidal neurons (Leuner et al., 2013). Therefore, the long-lasting antidepressant effects of Hyp + Lan and its impact on dendritic spine morphology could depend on the molecular synergism between NMDAR antagonism and TRPC6 receptor agonism.

In summary, we have shown that CRS + ZnD mice have different phenotypes than mice subjected to CRS alone. Discrepancies between these two groups are especially pronounced in their responses to typical and atypical antidepressant drugs. The findings of the present study show that the classic antidepressant IMI and atypical antidepressant compounds such as ketamine and Ro could not reverse the behavioral disturbances in CRS + ZnD animals. In contrast, ketamine and IMI showed antidepressant efficacies in the CRS group. The lack of antidepressant activity of ketamine is associated lower hippocampal and cortical Zn levels in CRS + ZnD animals; this suggests that adequate dietary Zn may be an essential factor in the antidepressant response of ketamine. Moreover, CRS + ZnD mice had lower hippocampal and cortical ketamine and (2R,6R;2S,6S)-HNK than the control and CRS animals, suggesting pharmacokinetic disturbances in mice with dietary Zn restriction. The CRS + ZnD group also exhibited more severe changes in the morphology of the hippocampal dendritic spines, which were not reversed by ketamine treatment, and only Hyp + Lan was effective under these experimental conditions. Since Hyp induces Zn-ion redistribution, it may work as a pharmacological intervention in patients with abnormal or low Zn levels. However, this issue requires further investigation wherein Hyp is co-administered with other antidepressant agents. Since dietary Zn restriction induces poor responses to atypical and common antidepressants, a well-balanced diet containing adequate Zn or Zn supplementation can be considered to augment antidepressant pharmacotherapy.

The present study has some limitations in that it is descriptive and does not explore the detailed mechanisms underlying the lack of antidepressant responses to IMI and ketamine in CRS + ZnD animals. Furthermore, we used only single doses (10 mg/kg each of IMI and ketamine) of the selected compounds; hence, any conclusions should be limited to these doses. This is especially important for ketamine because its hippocampal and cortical concentrations were lower than in the CRS and control animals. Thus, it is possible that administering higher doses of ketamine can overcome this issue. Based on previous studies of CRS and dietary Zn restriction, several other brain structures besides the Hp or FC may be involved. Therefore, complete understanding of the effects of combined CRS and ZnD would require further investigations into other brain regions.

## Data Availability

The raw data supporting the conclusion of this study will be made available by the authors without undue reservation.
